# Frailty index and all-cause and cause-specific mortality in Chinese adults: a prospective cohort study

**DOI:** 10.1016/S2468-2667(20)30113-4

**Published:** 2020-11-30

**Authors:** Junning Fan, Canqing Yu, Yu Guo, Zheng Bian, Zhijia Sun, Ling Yang, Yiping Chen, Huaidong Du, Zhongxiao Li, Yulong Lei, Dianjianyi Sun, Robert Clarke, Junshi Chen, Zhengming Chen, Jun Lv, Liming Li, China Kadoorie Biobank Collaborative Group

**Affiliations:** a Department of Epidemiology and Biostatistics, School of Public Health, Peking University, Beijing, China; b Peking University Institute of Environmental Medicine, Peking University, Beijing, China; c Peking University Health Science Center, and Key Laboratory of Molecular Cardiovascular Sciences, Ministry of Education, Peking University, Beijing, China; d Chinese Academy of Medical Sciences, Beijing, China; e Clinical Trial Service Unit and Epidemiological Studies Unit, Nuffield Department of Population Health, University of Oxford, Oxford, UK; f Medical Research Council Population Health Research Unit, Nuffield Department of Population Health, University of Oxford, Oxford, UK; g Noncommunicable Diseases Prevention and Control Department, Maiji Centre for Disease Control and Prevention, Tianshui, China; h China National Center for Food Safety Risk Assessment, Beijing, China

## Abstract

**Background:**

The fraily index is a useful proxy measure of accelerated biological ageing and in estimating all-cause and cause-specific mortality in older individuals in European and US populations. However, the predictive value of the frailty index in other populations outside of Europe and the USA and in adults younger than 50 years is unknown. We aimed to examine the association between the frailty index and mortality in a population of Chinese adults.

**Methods:**

In this prospective cohort study, we used data from the China Kadoorie Biobank. We included adults aged 30–79 years from ten areas (five urban areas and five rural areas) of China who had no missing values for the items that made up the frailty index. We did not exclude participants on the basis of baseline morbidity status. We calculated the follow-up person-years from the baseline date to either the date of death, loss to follow-up, or Dec 31, 2017, whichever came first, through linkage with the registries of China's Disease Surveillance Points system and local residential records. Active follow-up visits to local communities were done annually for participants who were not linked to any established registries. Causes of death from official death certificates were supplemented, if necessary, by reviewing medical records or doing standard verbal autopsy procedures. The frailty index was calculated using 28 baseline variables, all of which were health status deficits measured by use of questionnaires and physical examination. We defined three categories of frailty status: robust (frailty index ≤0·10), prefrail (frailty index >0·10 to <0·25), and frail (frailty index ≥0·25). The primary outcomes were all-cause mortality and cause-specific mortality in Chinese adults aged 30–79 years. We used a Cox proportional hazards model to estimate the associations between the frailty index and all-cause and cause-specific mortality, adjusting for chronological age, education, and lifestyle factors.

**Findings:**

512 723 participants, recruited between June 25, 2004, and July 15, 2008, were followed up for a median of 10·8 years (IQR 10·2–13·1; total follow-up 5 551 974 person-years). 291 954 (56·9%) people were categorised as robust, 205 075 (40·0%) people were categorised as prefrail, and 15 694 (3·1%) people were categorised as frail. Women aged between 45 years and 79 years had a higher mean frailty index and a higher prevalence of frailty than did men. During follow-up, 49 371 deaths were recorded. After adjustment for established and potential risk factors for death, each 0·1 increment in the frailty index was associated with a higher risk of all-cause mortality (hazard ratio [HR] 1·68, 95% CI 1·66–1·71). Such associations were stronger among younger adults than among older adults (p_interaction_<0·0001), with HRs per 0·1 increment of the frailty index of 1·95 (95% CI 1·87–2·03) for those younger than 50 years, 1·80 (1·76–1·83) for those aged 50–64 years, and 1·56 (1·53–1·59) for those 65 years and older. After adjustments, there was no difference between the sexes in the association between the frailty index and all-cause mortality (p_interaction_=0·75). For each 0·1 increment of the frailty index, the corresponding HRs for risk of death were 1·89 (95% CI 1·83–1·94) from ischaemic heart disease, 1·84 (1·79–1·89) from cerebrovascular disease, 1·19 (1·16–1·22) from cancer, 2·54 (2·45–2·63) from respiratory disease, 1·78 (1·59–2·00) from infection, and 1·78 (1·73–1·83) from all other causes.

**Interpretation:**

The frailty index is associated with all-cause and cause-specific mortality independent of chronological age in younger and older Chinese adults. The identification of younger adults with accelerated ageing by use of surrogate measures could be useful for the prevention of premature death and the extension of healthy active life expectancy.

**Funding:**

The National Natural Science Foundation of China, the National Key R&D Program of China, the Chinese Ministry of Science and Technology, the Kadoorie Charitable Foundation, and the Wellcome Trust.

## Introduction

Accelerated ageing occurs when biological age exceeds chronological age and is associated with a high risk of morbidity and mortality, and a reduced life expectancy.^1^ The identification of individuals whose biological age exceeds their chronological age could enable approaches to prevent premature death and extend healthy active life expectancy.

Several surrogate measures of biological age have been developed, including telomere length and epigenetic clocks.^2^ The frailty index, one of the widely used measures of biological age,^3^ is an established predictor of all-cause mortality in older (≥50 years) European and US populations.^4^, ^5^, ^6^ Previous studies have shown that the frailty index is a better predictor of all-cause mortality in white people than are measures of DNA methylation and chronological age among nonagenarians.^7^

**Research in context**

**Evidence before this study**

We searched PubMed for articles published from database inception to Dec 30, 2019, using a combination of terms: (“frailty” OR “frailty index” OR “frail” OR “frailty phenotype”) AND (“death” OR “mortality”). No restrictions on study type or language were implemented. We also found relevant studies by checking the reference lists of identified articles. We found well established evidence that frailty index predicts all-cause mortality. Most evidence came from studies done with European and US poplulations and older populations. Among younger adults who are at the lower end of the continuum for frailty status, whether the frailty index also functions as a surrogate measure for biological age, and differentiates between the risks of mortality in individuals of a similar chronological age, is unclear. Evidence regarding the association between the frailty index and cause-specific mortality in younger adults (<50 years) was also sparce.

**Added value of this study**

In this prospective cohort of more than half a million Chinese adults, we constructed a 28-item frailty index and found that women, older individuals (≥65 years), or those with unhealthy lifestyles had a higher prevalence of prefrailty and frailty. After adjustment for chronological age and other risk factors for death, the frailty index was associated with an increased risk of both all-cause and cause-specific mortality, with no clinically meaningful difference between women and men. Such associations were stronger among younger adults (<50 years) than among older adults (50–64 years and ≥65 years). To our knowledge, this is the first study to comprehensively analyse the association between accelerated ageing, as measured by the frailty index, and all-cause and cause-specific mortality in a large prospective cohort of Chinese adults.

**Implications of all the available evidence**

Despite being widely used in the field of geriatrics, the frailty index, which serves as a surrogate measure for biological age, seems to be a better predictor of mortality in younger adults (<50 years) than in older adults (≥50 years). Further research is needed to explore the use of these measures, which can be constructed by use of routine clinical examination and electronic health records, in the risk stratification of younger adults. The identification of those at risk might help to extend healthspan.

It is unclear whether the frailty index also functions as a surrogate measure for biological age and a predictor of the risk of adverse health outcomes among adults younger than 50 years who are at the lower end of the continuum of frailty status. Several studies from European populations have reported that the associations between the frailty index and all-cause mortality were somewhat stronger in younger than in older participants.^8^, ^9^, ^10^ Whether these findings apply to the Chinese population is unknown. Furthermore, studies on accelerated ageing and all-cause and cause-specific mortality in adults younger than 50 years are sparse.

In our study, we aimed to examine the predictive value of the frailty index in estimating all-cause and cause-specific mortality and compared the strength of such associations between Chinese adults of different age groups.

## Methods

### Study design and participants

In this prospective cohort study, we sourced data from the China Kadoorie Biobank. Details of the design and survey methods of the China Kadoorie Biobank have been described elsewhere.^11^ Briefly, the baseline survey was done between June 25, 2004, and July 15, 2008, in five urban areas (ie, Harbin, Qingdao, Suzhou, Liuzhou, and Haikou) and five rural areas (ie, Gansu, Henan, Sichuan, Zhejiang, and Hunan) across China. In the present analysis, we included participants aged 30–79 years who had no missing values for the items that made up the frailty index. We did not exclude participants on the basis of baseline morbidity status. We excluded two participants who had missing data for body-mass index (BMI). Long-term follow-up for mortality was done through linkage with the registries of China's Disease Surveillance Points system^12^ and local residential records. Causes of death from official death certificates were supplemented, if necessary, by reviewing medical records or doing standard verbal autopsy procedures. To minimise the potential for under-reporting, active follow-up visits to local communities were done annually for participants who were not linked to any established registries. All participants provided written informed consent. The study was approved by the Ethics Committee of the Chinese Center for Disease Control and Prevention (Beijing, China) and the Oxford Tropical Research Ethics Committee at the University of Oxford (Oxford, UK). The access policy and procedures are available online.

### Procedures

Data for sociodemographic characteristics (eg, age, sex, geographical location, and level of education), dietary and lifestyle factors (eg, tobacco smoking, alcohol consumption, physical activity, and intake of fresh fruits, vegetables, and red meat), sleeping habits, mental status (ie, moods and emotions), and personal and family medical history (eg, of heart attack, stroke, and cancer) were collected by trained staff using a laptop-based questionnaire. Physical measurements, including height, weight, waist and hip circumference, blood pressure, and lung function, were recorded by use of calibrated instruments and following standard procedures. A 10 mL non-fasting blood sample was collected for storage and on-site random testing of plasma glucose was done. More detailed information on other data and measurements collected, but not used for this study, are available online.

Information about physical activity in the past year was collected by asking participants about their usual type of activity and the duration of activity for each of the four domains of physical activity (ie, occupational, commuting, housework, and leisure time). The daily level of physical activity was calculated by multiplying the value of the metabolic equivalent task for a particular type of activity by the hours spent on that activity per day and then summing the metabolic equivalent task-hours for all activities.^13^ The possible answers for the frequency of dietary intake were daily, 4–6 days per week, 1–3 days per week, monthly, or rarely or never. We obtained the covariates from the baseline questionnaire.

We constructed the frailty index following a standard procedure.^14^ Deficits associated with health status were included in the frailty index if they met the following criteria: the deficit involves multiple body systems and a range of physiological areas; the prevalence of the deficit generally increases with age; the deficit is not nearly universal in middle-age; the deficit has a baseline prevalence of 0·5% or more in the population of the China Kadoorie Biobank; and the deficit has a missingness of 5·0% or less. We added the criteria of baseline prevalence and missingness to the standard procedure of construction of the frailty index. If two deficit variables were highly correlated (ie, if the Spearman's rank correlation coefficients were >0·4), the variable that had a higher correlation with age was included.^15^

After screening baseline self-reported or measured data, we selected 28 variables to use to calculate the frailty index, including medical conditions (based on self-reports of diagnosis by a doctor or physical measurements), symptoms, signs, and physical measurements (table 1, appendix 2 pp 2–4). Each deficit was dichotomised or mapped into the 0·00–1·00 interval, with 0·00 indicating the absence of a deficit (the healthiest state) and 1·00 indicating the maximal expression of the deficit (the unhealthiest state). The frailty index was calculated for each participant as the number of deficits present in a person divided by the 28 deficits considered. Following the consensus on the construction of the frailty index, we did not assign weight to the variables that were related to each other. The nature of the deficits included in the frailty index might be less important than the number of deficits.^16^ The frailty index is a continuous variable that ranged from 0·00 to 1·00, with a higher value indicating a worse, frailer status. With reference to previous studies,^3^, ^17^, ^18^ we further categorised the frailty index into three levels of frailty: robust (frailty index ≤0·10), prefrail (frailty index >0·10 to <0·25), and frail (frailty index ≥0·25). The cutoff point of 0·10 was roughly equivalent to three deficits and the cutoff point of 0·25 was roughly equivalent to seven deficits.

**Table 1:** List of 28 variables included in the frailty index

	**Definition according to baseline self-report, physical measurements, or both**	**Coding of variables**
1	Self-reported diagnosis of hypertension by a doctor, self-reported use of antihypertension drugs, systolic blood pressure measured to be ≥140 mm Hg, or diastolic blood pressure measured to be ≥90 mm Hg	Yes=1·00; no=0·00
2	Self-reported diagnosis of heart disease by a doctor	Yes=1·00; no=0·00
3	Self-reported diagnosis of stroke or transient ischaemic attack by a doctor	Yes=1·00; no=0·00
4	Self-reported diagnosis of emphysema or chronic bronchitis by a doctor	Yes=1·00; no=0·00
5	Self-reported diagnosis of tuberculosis by a doctor	Yes=1·00; no=0·00
6	Self-reported diagnosis of asthma by a doctor	Yes=1·00; no=0·00
7	Self-reported diagnosis of peptic ulcer by a doctor	Yes=1·00; no=0·00
8	Self-reported diagnosis of gallstone disease, with or without cholecystitis, by a doctor	Yes=1·00; no=0·00
9	Self-reported diagnosis of rheumatoid arthritis by a doctor	Yes=1·00; no=0·00
10	Self-reported diagnosis of fracture by a doctor	Yes=1·00; no=0·00
11	Self-reported diagnosis of neurasthenia by a doctor	Yes=1·00; no=0·00
12	Self-reported diagnosis of diabetes, fasting blood glucose measured to be ≥7·0 mmol/L, or random blood glucose measured to be ≥11·1 mmol/L	Yes=1·00; no=0·00
13	Self-reported diagnosis of cancer by a doctor	Yes=1·00; no=0·00
14	Self-reported diagnosis of chronic kidney disease by a doctor	Yes=1·00; no=0·00
15	If you were walking on level ground with other healthy people of the same age, would you usually become short of breath or slow down because of chest discomfort?	Yes=1·00; no=0·00
16	During the past month, did you have any of the following for ≥3 days per week: (1) taking >30 min to fall asleep after going to bed or waking up in the middle of the night; (2) waking up early and not being able to go back to sleep; or (3) having difficulty staying alert while at work, eating, or meeting people during the daytime?	Yes=1·00; no=0·00
17	How often do you have bowel movements each week?	<3 times per week=1·00; other=0·00
18	During the past 12 months, did you have pain or discomfort in your body lasting ≥3 months that interfered with your life?	Yes=1·00; no=0·00
19	During the past 12 months, have you developed a frequent cough?	Yes, for ≥3 months=1·00; yes, for <3 months=0·50; no=0·00
20	Do you brush your teeth rarely or never, or have false teeth?	Yes=1·00; no=0·00
21	Physical activity in the past 12 months, including the usual type and duration of activities in occupational, commuting, domestic, and leisure time-related domains	Lowest quintile stratified by sex=1·00; other=0·00
22	During the past 12 months, have you lost weight (≥2·5 kg) despite not trying to intentionally lose weight?	Yes=1·00; no=0·00
23	During the past 12 months, did you feel much sadder, or more depressed, than usual for ≥2 weeks?	Yes=1·00; no=0·00
24	How is your current general health status?	Poor=1·00; fair=0·50; good=0·25; excellent=0·00
25	Body-mass index (kg/m^2^)*	<18·5 or ≥28·0=1·00; ≥24·0 and <28·0=0·50; ≥18·5 and <24·0=0·00
26	Waist circumference (cm) to hip circumference ratio	≥0·95 for men or ≥0·90 for women=1·00; ≥0·90 and <0·95 for men or ≥0·85 and <0·90 for women=0·50; <0·90 for men or <0·85 for women=0·00
27	Measured heart rate, beats per min	<60 or >100=1·00; ≥60 and ≤100=0·00
28	The ratio of forced expiratory volume in 1 s to the forced vital capacity measured to be <0·7	Yes=1·00; no=0·00

* Body-mass index was calculated by dividing the weight (kg) of an individual by their height (m^2^).

### Outcomes

The primary outcomes were all-cause mortality and cause-specific mortality in Chinese adults aged 30–79 years. Causes of death were classified by use of the 10th revision of the International Classification of Diseases (eg, I20–25 for ischaemic heart diseases, I60–69 for cerebrovascular diseases, C00–97 for cancer, J00–99 for diseases of the respiratory system, and A00–B99 for infections).

### Statistical analysis

Baseline characteristics were presented by three categories of frailty status (ie, robust, prefrail, and frail) as means (SD) for continuous variables or percentages for categorical variables, with adjustment for age, sex, and study area. We plotted the mean frailty index and the prevalence of frailty by age and sex to examine the associations of the frailty index with age. We used multinomial logistic regression to calculate the baseline prevalence of prefrail and frail statuses by baseline characteristics, with adjustment for age, sex, and study area.

We calculated the follow-up person-years from the baseline date to either the date of death, loss to follow-up, or Dec 31, 2017, whichever came first. We used a Kaplan-Meier survival curve to compare survival probabilities after baseline enrolment between different groups of frailty status stratified by baseline age. We used a Cox proportional hazards model to estimate the associations between the frailty index, included in the model as a continuous or a categorical variable, and all-cause and cause-specific mortality. The model used age as the underlying timescale and was stratified jointly by baseline age (with the groups, 30–34 years, 35–39 years, 40–44 years, 45–49 years, 50–54 years, 55–59 years, 60–64 years, 65–69 years, 70–74 years, and 75–79 years), sex (two groups), and study area (ten groups). Multivariable models were adjusted for age, education level, tobacco smoking (non-smoker; former smoker who had stopped for reasons other than illness; and current smoker or former smoker who had stopped because of illness [from one to 14 cigarettes or equivalent per day; 15–24 cigarettes or equivalent per day; and ≥25 cigarettes or equivalent per day]), alcohol consumption (never weekly drinker; former weekly drinker; weekly, but not daily, drinker; <15 g per day of pure alcohol; 15–29 g per day of pure alcohol; 30–59 g per day of pure alcohol; and ≥60 g per day of pure alcohol), intake frequency of fresh fruits, vegetables, and red meat (days per week), and, only in the corresponding cause-specific analyses, family history of heart attack, stroke, or cancer. Stratified analyses were done by age at baseline (<50 years, 50–64 years, and ≥65 years), sex (men and women), and region (urban and rural). We tested for interactions using the likelihood ratio test, which involved comparing models with and without interaction terms.

To understand the role that the prevalence of major chronic diseases, such as heart disease, stroke, cancer, chronic obstructive pulmonary disease (COPD), and diabetes, has in the association between the frailty index and overall mortality and cause-specific mortality, we excluded individuals, either separately or together, with one or more of these chronic diseases at baseline from the analysis. The corresponding items were naturally removed from the frailty index. To eliminate the effect of smoking on health status, we excluded current smokers and former smokers (at the time the baseline survey was done) who had quit because of illnesss. We also excluded those who had died during the first 5 years of follow-up to minimise any potential reverse causality.

We did all statistical analyses using Stata version 15.0 and plotted graphs using R version 3.5.3. All p values were two-sided and the level of statistical significance was defined as p less than 0·05.

### Role of the funding source

The funders had no role in study design, data collection, data analysis, data interpretation, or writing of the report. The corresponding author had full access to all the data in the study and had final responsibility for the decision to submit for publication.

## Results

Between June 25, 2004, and July 15, 2008, the China Kadoorie Biobank collected data from 512 725 adults from ten areas of China.^11^ In this prospective cohort study, we excluded two participants who had missing data for BMI and included 512 723 adults aged 30–79 years who were followed up for a median of 10·8 years (IQR 10·2–13·1; total follow-up 5 551 974 person-years). The baseline characteristics of the study participants by frailty status can be found in table 2.

**Table 2:** Baseline characteristics of the study participants by frailty status

		**Robust (frailty index ≤0·10; n=291 954)**	**Prefrail (>0·10 to <0·25; n=205 075)**	**Frail (frailty index ≥0·25; n=15 694)**	**Total (n=512 723)**
**Sociodemographics**					
Sex					
	Men*	121 947 (58·0%)	82 619 (39·3%)	5636 (2·7%)	210 202
	Women*	170 007 (56·2%)	122 456 (40·5%)	10 058 (3·3%)	302 521
Age, years		48·9 (9·7)	55·8 (10·4)	61·7 (9·2)	52·0 (10·7)
	<50 years*	167 335 (72·6%)	61 239 (26·6%)	1816 (0·8%)	230 390
	50–64 years*	102 747 (49·5%)	97 532 (47·0%)	7234 (3·5%)	207 513
	≥65 years*	21 872 *(*29·2%)	46 304 (61·9%)	6644 (8·9%)	74 820
Place of residence					
	Urban area	127 309 (43·6%)	91 827 (44·8%)	7056 (45·0%)	226 192 (44·1%)
	Rural area	164 645 (56·4%)	113 248 (55·2%)	8638 (55·0%)	286 531 (55·9%)
Schooling					
	No formal school	43 418 (17·9%)	46 712 (19·2%)	5048 (20·1%)	95 178 (18·6%)
	Primary school (ages 6–11 years)	88 216 (31·7%)	714 44 (33·1%)	5527 (32·9%)	165 187 (32·2%)
	Middle school (ages 12–14 years)	90 382 (28·2%)	51 487 (28·5%)	3005 (29·0%)	144 874 (28·3%)
	High school (ages 15–17 years)	50 130 (15·8%)	25 926 (14·2%)	1452 (12·8%)	77 508 (15·1%)
	College or university	19 808 (6·4%)	9506 (5·1%)	662 (5·2%)	29 976 (5·8%)
**Medical history**†‡					
Hypertension		52 531 (20·2%)	116 194 (52·8%)	11 863 (67·2%)	180 588 (35·2%)
Heart disease		1335 (0·6%)	10 311 (4·2%)	3826 (16·3%)	15 472 (3·0%)
Stroke or transient ischaemic attack		516 (0·2%)	6079 (2·4%)	2289 (9·3%)	8884 (1·7%)
Emphysema or bronchitis		1593 (0·6%)	8492 (4·0%)	3203 (18·7%)	13 288 (2·6%)
Tuberculosis		1876 (0·7%)	4755 (2·2%)	1028 (5·3%)	7659 (1·5%)
Asthma		372 (0·1%)	1641 (0·9%)	793 (6·2%)	2806 (0·5%)
Peptic ulcer		5774 (1·9%)	12 490 (6·2%)	1751 (11·9%)	20 015 (3·9%)
Gallstone diseases		7486 (2·5%)	20 116 (10·0%)	3395 (21·0%)	30 997 (6·0%)
Rheumatoid arthritis		2041 (0·7%)	6996 (3·3%)	1587 (9·3%)	10 624 (2·1%)
Fracture		11 172 (3·7%)	21 697 (10·9%)	2575 (18·8%)	35 444 (6·9%)
Neurasthenia		877 (0·3%)	3742 (1·8%)	1080 (7·0%)	5699 (1·1%)
Diabetes		3567 (1·3%)	21 946 (10·0%)	4787 (26·7%)	30 300 (5·9%)
Cancer		420 (0·2%)	1807 (0·8%)	351 (1·7%)	2578 (0·5%)
Chronic kidney disease		1700 (0·6%)	4804 (2·5%)	1071 (7·0%)	7575 (1·5%)
**Symptoms and signs**†					
Short of breath or slows down when walking		2661 (0·9%)	21 647 (10·9%)	8302 (53·1%)	32 610 (6·4%)
Sleep problem		22 511 (7·7%)	54 903 (26·8%)	8452 (53·7%)	85 866 (16·7%)
Abnormal frequency of bowel movement		7869 (2·6%)	12 611 (6·5%)	2178 (15·1%)	22 658 (4·4%)
Body pain or discomfort		854 (0·3%)	3694 (2·1%)	1012 (8·0%)	5560 (1·1%)
Cough frequently		3745 (1·2%)	12 380 (6·6%)	3445 (25·6%)	19 570 (3·8%)
Dental problem		11 885 (6·1%)	32 592 (12·0%)	4603 (17·0%)	49 080 (9·6%)
Low level of physical activity		26 252 (10·8%)	66 638 (28·2%)	9450 (48·6%)	102 340 (20·0%)
Unintentional weight loss in the past 12 months		15 248 (4·9%)	32 726 (17·2%)	5092 (37·1%)	53 066 (10·3%)
Have felt sad or depressed in the past 12 months		2823 (0·9%)	10 172 (5·9%)	2543 (23·0%)	15 538 (3·0%)
Self-reported poor health status		8413 (2·8%)	35 391 (17·6%)	9287 (59·9%)	53 091 (10·4%)
**Physical measurement**†					
BMI <18·5 kg/m^2^ or ≥28·0 kg/m^2^		21 519 (7·2%)	49 458 (24·8%)	5724 (38·6%)	76 701 (15·0%)
WHR ≥0·95 for men or ≥0·90 for women		48 336 (17·4%)	97 182 (45·8%)	9633 (58·0%)	155 151 (30·3%)
Heart rate <60 beats per min or >100 beats per min		12 058 (3·9%)	24 688 (13·1%)	3387 (25·7%)	40 133 (7·8%)
FEV_1_:FVC <0·7		5999 (2·4%)	17 767 (7·5%)	3921 (18·3%)	27 687 (5·4%)
**Frailty index**					
Mean frailty index					
	Men	0·055 (0·028)	0·148 (0·035)	0·283 (0·039)	0·098 (0·062)
	Women	0·055 (0·028)	0·149 (0·036)	0·285 (0·040)	0·101 (0·065)
Frailty index at the 99th centile					
	Men	0·098	0·232	0·411	0·286
	Women	0·098	0·232	0·429	0·286

Data are n (%) or mean (SD), unless otherwise stated. Baseline characteristics were adjusted for age, sex, and study area, except in the cases where age, sex, or study area was the independent variable. BMI=body-mass index. FEV_1_=forced expiratory volume in 1 s. FVC=forced vital capacity. WHR=waist to hip ratio.

* The percentages for these rows were calculated by use of the total number of participants in different sex or age groups as the denominator.

† The percentages for these categories represent the proportion of the deficit in different frailty statuses.

‡ The statuses of hypertension and diabetes were based on self-reported diagnosis by a doctor and baseline measurements. Other medical histories were based on self-reports of diagnoses by doctors.

The frailty index showed a right-skewed distribution towards older age (appendix 2 p 5). The mean frailty index across all participants was 0·099 (SD 0·064). 291 954 (56·9%) people were categorised as robust, 205 075 (40·0%) people were categorised as prefrail, and 15 694 (3·1%) people were categorised as frail (table 2). Overall, the maximum score of the frailty index in any individual was 0·589 in women and 0·652 in men, consistent with an empirical limit to the frailty index of about 0·7.

**Figure 1:**
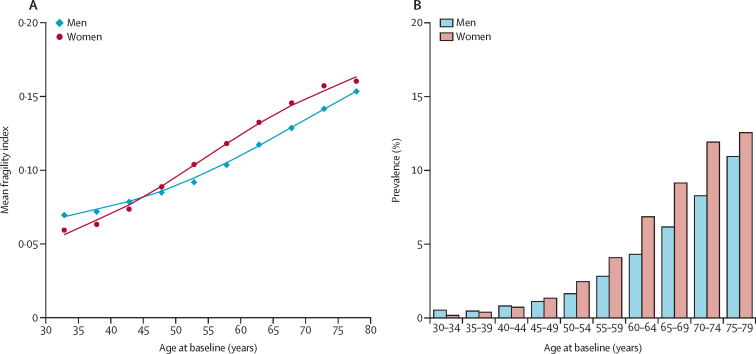
Mean frailty index and frailty prevalence by age and sex (A) The datapoints represents the mean value of the frailty index per each 5-year age group and the lines represents the fitted curve of the frailty index. (B) The histogram represents the prevalence of frailty per each 5-year age group.

**Figure 2:**
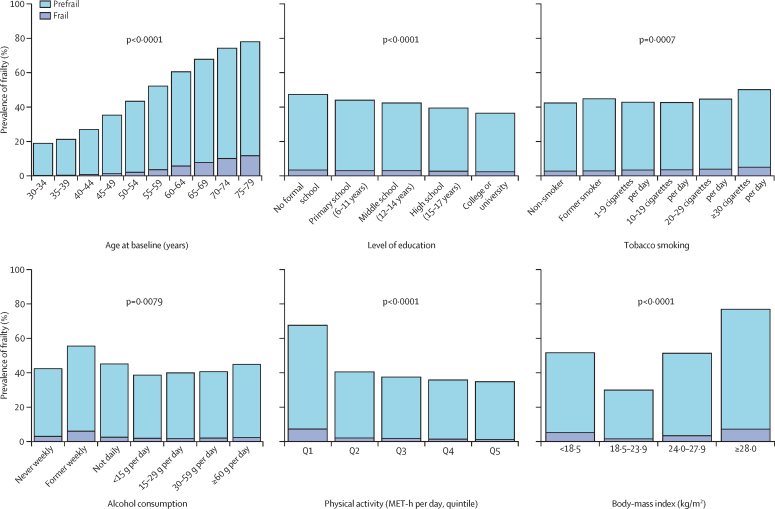
The prevalence of frailty by baseline characteristics The prevalence of frailty was adjusted for age, sex, and study area, except for prevalence by age groups (panel A), which was not adjusted for any variable. Former smokers who had stopped smoking because of illness were classified as current smokers. We report the p values for trend by age, education level, tobacco smoked among daily smoker, pure alcohol consumed among daily drinker, and physical activity. MET-h=metabolic equivalent of task-hours.

Both the mean frailty index and the prevalence of frailty increased with age. The prevalence of frailty increased from 1816 (0·8%) in 230 390 people aged <50 years to 7234 (3·5%) in 207 513 people aged 50–64 years, and to 6644 (8·9%) in 74 820 people aged 65 years and older. Between age 45 years and 79 years, women had a higher mean frailty index and a higher prevalence of frailty than did men (table 2; figure 1). The prevalence of frailty and prefrailty was higher in participants who were less educated; daily, heavy drinkers of alcohol (≥60 g of pure alcohol per day) or non-daily drinkers of alcohol (ie, the never weekly, former weekly, and not daily drinkers); physically inactive; underweight (BMI <18·5 kg/m^2^) or overweight (BMI ≥24·0 kg/m^2^), or who had lower intakes of fresh fruits (figure 2, appendix 2 pp 6–7).

During follow-up, 49 371 deaths were documented: 7488 (15·2%) deaths from ischaemic heart diseases, 10 933 (22·1%) from cerebrovascular diseases, 15 750 (31·9%) from cancer, 4652 (9·4%) from respiratory diseases, 629 (1·3%) from infections, and 9919 (20·1%) from all other causes. Based on the Kaplan-Meier curves of survival probability for participants during follow-up, the frail participants at baseline, regardless of age, had the lowest survival probabilities compared with those participants who were classified as robust or prefrail (appendix 2 pp 8–9). The difference between the groups of frailty status in survival probability over time increased with baseline age.

**Figure 3:**
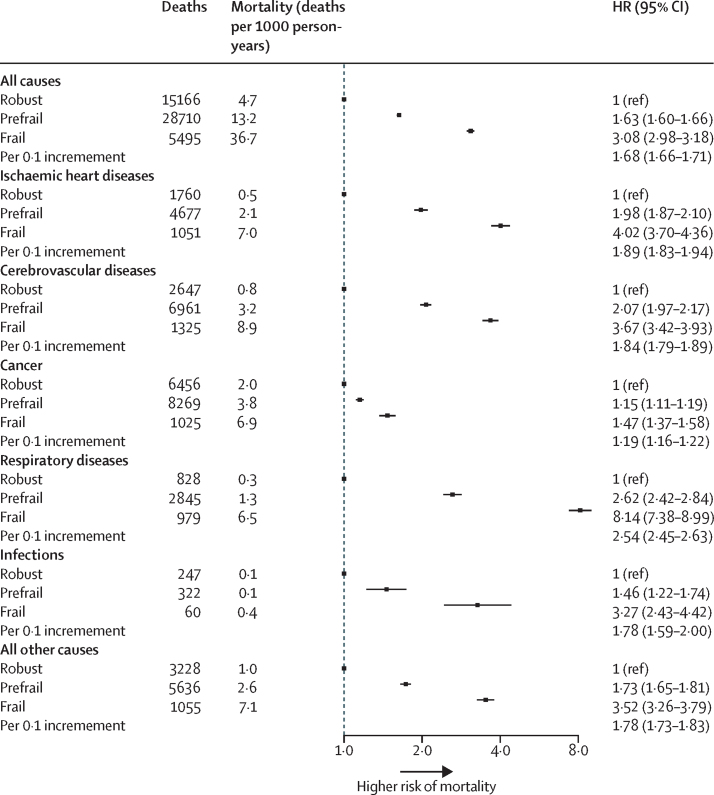
The association of frailty status with all-cause and cause-specific mortality All models were stratified by age, sex, and study area. Multivariate models were adjusted for age, education level, tobacco smoking, alcohol, intake frequency of fresh fruits, vegetables, and red meat, and family disease history of heart attack, stroke, and cancer in corresponding cause-specific analyses. HR=hazard ratio.

**Figure 4:**
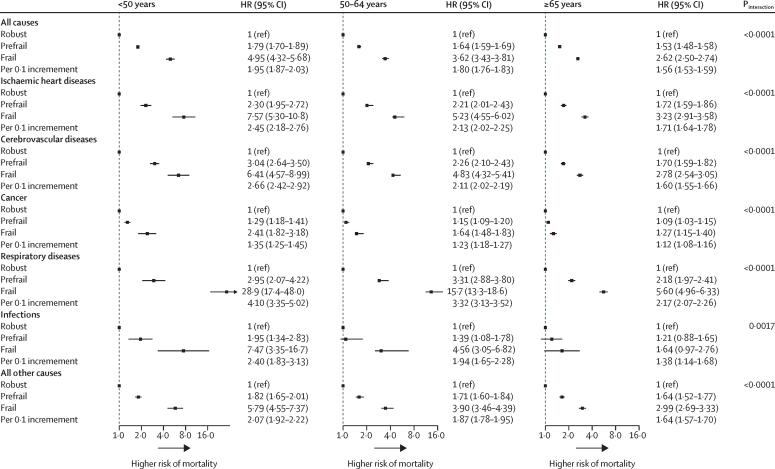
The association of frailty status with all-cause and cause-specific mortality among different age groups All models were stratified by sex and study area. Multivariate models were adjusted for age, education level, tobacco smoking, alcohol, intake frequency of fresh fruits, vegetables, and red meat, and family disease history of heart attack, stroke, and cancer in corresponding cause-specific analyses. HR=hazard ratio.

For all-cause mortality, compared with robust participants, prefrail participants had a higher risk of death, as did frail participants (figure 3). The overall adjusted HR for all-cause mortality per 0·1 increment in the frailty index was 1·68 (95% CI 1·66–1·71; figure 3). Such associations were stronger among younger adults (<50 years) than among older adults (50–64 years and ≥65 years; p_interaction_<0·0001; figure 4). There was no difference between the sexes in the association between the frailty index and all-cause mortality (p_interaction_=0·75; appendix 2 pp 10–11). However, the association was significantly different for participants who lived in rural areas compared with those who lived in urban areas, with participants living in rural locations generally having higher HRs associated with frailty and pre-frailty than participants living in urban locations (appendix 2 pp 12–13).

A graded increase in the risk of several causes of death was also observed across the prefrail and frail groups (figure 3). For each 0·1 increment of the frailty index, the corresponding HRs for risk of death were 1·89 (95% CI 1·83–1·94) from ischaemic heart disease, 1·84 (1·79–1·89) from cerebrovascular disease, 1·19 (1·16–1·22) from cancer, 2·54 (2·45–2·63) from respiratory disease, 1·78 (1·59–2·00) from infection, and 1·78 (1·73–1·83) from all other causes. Stratified analyses by age showed that the association of the frailty index with cause-specific mortality persisted in all three age groups, but was larger for younger participants (aged <50 years) than for older participants (aged 50–64 years and ≥65 years; figure 4). Although there was a significant difference in the association of the frailty index with the risk of mortality from ischaemic heart diseases, cancer, and other causes between men and women, the difference in the size of the association was not clinically meaningful (appendix 2 pp 10–11). The association of the frailty index with risk of death from respiratory diseases was stronger for participants who lived in rural areas than those who lived in urban areas (appendix 2 pp 12–13).

After excluding participants with heart disease, stroke, cancer, COPD, or diabetes at baseline, the association of the frailty index with all-cause mortality and cause-specific mortality showed small to moderate changes (appendix 2 p 14). The most apparent attenuation of this association occurred in mortality from respiratory diseases after participants with COPD at baseline were removed from the analyses. After removing all participants who had any of the five aforementioned diseases at baseline, the association of the frailty index with all-cause and cause-specific mortality was moderately reduced, but persisted. The HR for all-cause mortality per 0·1 increment in the reduced frailty index was 1·36 (95% CI 1·34–1·38). Exclusion of people from the analyses who were current smokers or died during the first 5 years of follow-up only changed the HRs slightly.

## Discussion

In this prospective cohort of more than half a million Chinese adults, we constructed a 28-item frailty index and found that women, older individuals (≥65 years), or those with unhealthy lifestyles, had a higher prevalence of prefrailty and frailty. After adjustment for chronological age and other risk factors for death, accelerated ageing, as indicated by the frailty index, was associated with an increased risk of both all-cause mortality and cause-specific mortality, with the highest risk from respiratory diseases and the lowest risk from cancer. Importantly, an increase in the frailty index conferred a higher risk of mortality among younger participants (<50 years) than it did among older participants (≥50 years).

The frailty index measure generated in our study had predicted characteristics, including the right-skewed distribution to older age, the limit of about 0·7, and the higher levels of frailty in women than in men, consistent with the results of previous studies.^14^, ^15^, ^19^ The prevalence of frailty was 8·9% in participants aged 65 years and older. One meta-analysis of 14 studies with a total of 81 258 participants reported similar results to the present study, with a pooled frailty prevalence of 10% (95% CI 8–12) among Chinese adults in the community aged 65 years and older.^20^

The present study comprehensively analysed the association between accelerated ageing, as measured by the frailty index, and all-cause and cause-specific mortality in a large prospective cohort in China. The identification of younger adults (<50 years) with accelerated ageing by use of surrogate measures is meaningful for the prevention of premature death and the extension of healthy active life expectancy. The strengths of our study include its prospective design and the inclusion of a geographically dispersed and socioeconomically diverse population. The inclusion of 230 390 adults younger than 50 years with a median follow-up of 10 years allowed a thorough analysis of cause-specific mortality in younger adults. The use of a continuous frailty index as a surrogate for biological age is more sensitive in younger adults who are at the lower end of the frailty spectrum. We constructed the frailty index using both self-reported information and objective measured traits, which has been suggested to be more robust than using mostly subjective health measures.^21^ Furthermore, it is rare for the China Kadoorie Biobank to have missing data.

Our study also had some limitations. First, frailty was assessed only at one timepoint at baseline. The frailty status might have changed during follow-up, leading to non-differential misclassification that is more likely to bias the association towards the null hypothesis. Second, we did not collect and incorporate functional items, such as activities of daily living or instrumental activities of daily living, into the frailty index, which could have resulted in an incomplete picture of age-related change. Nevertheless, the frailty index appears to be insensitive to the choice of particular items.^22^ Third, information bias arising from the self-reporting of diseases and symptoms cannot be fully excluded. Finally, the China Kadoorie Biobank, being a prospective cohort, was not designed to be representative of the general population in China. Therefore, caution must be taken in generalising our findings to the broader Chinese population.

Previous studies have shown that a higher frailty index is associated with a greater relative risk of all-cause mortality and that the associations attenuate with age.^8^, ^9^, ^10^ Findings from a study that followed up 1477 Swedish adults for 30 years showed that the association between a 42-item frailty index and all-cause mortality was stronger among women younger than 65 years (HR per increase in one deficit 1·11, 95% CI 1·07–1·17) than among women 65 years and older (1·07, 1·04–1·10).^10^ In a study that used UK Biobank data from 500 336 participants aged 40–69 years with a follow-up of 10 years, the HRs per 0·1 increment of the frailty index for all-cause mortality were 1·87 (95% CI 1·74–2·00) for those younger than 50 years, 1·77 (1·70–1·83) for those aged 50–59 years, 1·60 (1·55–1·66) for those aged 60–64, and 1·59 (1·54–1·64) for those 65 years and older.^8^ Our study expanded on these findings by showing age differences for the associations of frailty index with mortality risk from ischaemic heart disease, cerebrovascular disease, respiratory disease, and infection. Such findings suggest that more research into accelerated ageing in younger populations and the development of relevant screening and intervention programmes are warranted.

A meta-analysis of five studies reported that women had a higher level of frailty across the age range, but had lower mortality at any given level of frailty or age than men, supporting a well described male–female health–survival paradox.^23^ However, one study in Sweden, together with the present findings, did not find differences between the sexes for the associations between the frailty index and mortality.^9^ Analysis of data from the UK Biobank indicated that the risk of mortality associated with an increased frailty index was higher in men (HR 1·72, 95% CI 1·68–1·76) than in women (1·56, 1·51–1·60).^8^ Conversely, data from a Swedish cohort with a 30 year follow-up showed the opposite; women had a higher effect size for the frailty-mortality association (HR 1·08, 95% CI 1·06–1·11) than men did (1·04, 1·01–1·07).^10^ As far as we are aware, there is no research similar to ours on the urban–rural difference in the association of the frailty index with mortality. Our study showed that, despite the risk of certain causes of death being significantly different between participants living in urban versus rural areas, the frailty index was an important predictor of mortality in both groups.

Previous studies on frailty and cause-specific mortality were limited by including only adults aged 50 years or older, or populations with a narrow age range. Our research had findings consistent with previous studies regarding the associations between prefrailty or frailty and the increased risk of mortality from ischaemic heart disease and stroke.^4^, ^9^, ^24^, ^25^ Two prospective cohort studies from Sweden reported no association between frailty and mortality from cancer.^9^, ^10^ The authors explained that the frailty level might be a valid predictor of cancer mortality only among those who already have cancer, a conclusion supported by other studies.^26^, ^27^, ^28^ However, in our study, the frailty index predicted mortality from cancer for more than 10 years after the baseline surey in both younger (<50 years) adults and older (≥50 years) adults, most of whom did not have cancer at the time of the assessment of frailty index. The association between the frailty index and the risk of death from cancer remained even after we excluded participants with cancer at baseline or those who died during the first 5 years of follow-up. Findings on cancer mortality warrant further confirmation because of inconsistencies between study results. Consistent with our results, studies have shown that frailty is associated with a higher likelihood of developing respiratory impairment,^29^ and an increased risk of respiratory^4^, ^9^, ^29^ and infection-related mortality.^4^ In our study, the association between the frailty index and respiratory mortality was considerably attenuated, but remained strong, after removing participants with COPD at baseline, indicating that the association was only partly driven by those who already had COPD. We also did similar sensitivity analyses by excluding participants with other major chronic diseases and some items that constituted the frailty index. The association between the frailty index and all-cause and cause-specific mortality remained strong, suggesting that the observed associations did not depend on the existence of any severe disease and that the frailty index is a robust surrogate measure for predicting the risk of death.

In this large, prospective cohort study of the Chinese population, we showed strong associations between the frailty index and the risk of all-cause and cause-specific mortality in both younger and older adults, even after adjusting for chronological age. The frailty index was a better predictor of mortality in younger adults (aged <50 years) than in older adults (aged ≥50 years). Despite the low prevalence of frailty in younger adults, a third of these adults were prefrail and had a significantly increased risk of death compared with adults who were classified as robust. Further research is needed to explore the use of these measures, which can be constructed by use of routine clinical examination and electronic health records, in the risk stratification of younger adults. The identification of those at risk might help to extend healthy active life expectancy. A data-driven approach is also an option in the creation of surrogate measures of biological age.

## Supplementary Materials

Chinese translation of the abstract
