# Supplementary material for: Frailty index and all-cause and cause-specific mortality in Chinese adults: a prospective cohort study
Source: Lancet Public Health. 2020 Nov 30;5(12):e650–60. doi: 10.1016/S2468-2667(20)30113-4 (PMC7708389; doi:10.1016/S2468-2667(20)30113-4)
Supplement: Chinese translation of the abstract [file mmc1.pdf]

THE LANCET
Public Health

## Supplementary appendix 1

This translation in Chinese was submitted by the authors and we reproduce it as supplied. It has not been peer reviewed. *The Lancet*'s editorial processes have only been applied to the original in English, which should serve as reference for this manuscript.

Supplement to: Fan J, Yu C, Guo Y, et al. Frailty index and all-cause and cause-specific mortality in Chinese adults: a prospective cohort study. *Lancet Public Health* 2020; **5:** e650–60.

**此简体中文译文由作者提交，我方按照提供的版本刊登。此译文并未经过同行审阅。医学期刊《柳叶刀》的编辑流程仅适用于英文原稿，英文原稿应作为此手稿的参考。**

**中国成人衰弱指数与全死因和死因别死亡的关联研究：一项前瞻性队列研究**

**摘要**

**背景：**在欧洲和美国的老年人群中，衰弱指数（frailty index）可作为生物学衰老加速的一个代理指标，估计全死因和死因别死亡风险。但是，在欧洲和美国以外的其他人群以及 50 岁以下的成年人中，衰弱指数的预测价值尚不清楚。本研究旨在检验中国成年人群中衰弱指数与死亡风险之间的关联。

**方法：**这项前瞻性队列研究使用中国慢性病前瞻性研究（China Kadoorie Biobank）的数据。分析中纳入来自中国 10 个地区（5 个城市地区和 5 个农村地区）的 30-79 岁成年人，且衰弱指数的构成条目没有缺失者。我们没有根据基线患病状态排除研究对象。随访人年的计算是从基线调查日期到死亡、失访或 2017 年 12 月 31 日（以最早发生日期为准）。随访结局是通过与中国疾病监测点系统和当地居民记录建立链接。对于那些未能与任何登记系统链接的研究对象，我们每年会主动与当地社区联系，获取相关信息。死因由死亡证获得，必要时通过检查病历或进行标准的死因推断来补充。衰弱指数由 28 个基线变量计算；这些变量都是反映个体的健康状况缺陷，通过问卷调查或体格检查获得。我们将衰弱状态分为三类：健康（衰弱指数≤0·10）、衰弱前期（衰弱指数>0·10 且<0·25）和衰弱（衰弱指数≥0·25）。主要的研究结局是 30-79 岁中国成年人的全死因和死因别死亡。我们使用 Cox 比例风险模型来估计衰弱指数与全死因和死因别死亡之间的关联，模型中同时调整实际年龄、受教育程度和多种生活方式因素。

**结果：**研究纳入 512723 名研究对象。基线募集于 2004 年 6 月 25 日至 2008 年 7 月 15 日，随访年限的中位数为 10·8 年（四分位间距：10·2-13·1 年；总人年数：5551974 人年）。291954（56·9%）名研究对象被归类为"健康"，205075（40·0%）名研究对象被归类为"衰弱前期"，15694（3·1%）名研究对象被归类为"衰弱"。45-79 岁人群中，女性的平均衰弱指数和衰弱率均高于男性。随访期间，49371 人死亡。在调整已知和潜在的混杂因素后，随衰弱指数增加，全死因死亡的风险增高；衰弱指数每增加 0·1 个单位，风险比[HR]为 1·68（95% CI 1·66-1·71）。低年龄组成年人中的此关联强度高于高年龄组（交互作用 P 值<0·0001）。衰弱指数每增加 0·1 个单位，在<50 岁年龄组的 HR 值为 1·95（95% CI 1·87-2·03），在 50-64 岁年龄组的 HR 值为 1·80（95% CI 1·76-1·83），在≥65 岁年龄组的 HR 值为 1·56（95% CI 1·53-1·59）。衰弱指数与全死因死亡的关联在男性和女性之间没有差异（交互作用 P 值=0·75）。衰弱指数每增加 0.1 个单位，各死因别死亡的 HRs（95%

CIs）分别为：缺血性心脏病死亡 1·89（1·83-1·94），脑血管疾病死亡 1·84（1·79-1·89），恶性肿瘤死亡 1·19（1·16-1·22），呼吸系统疾病死亡 2·54（2·45-2·63），感染死亡 1·78（1·59-2·00），其他死因 1·78（1·73-1·83）。

**解释：**在中年和老年中国人群中，衰弱指数独立于实际年龄，与全死因死亡和死因别死亡风险存在关联。利用代理指标（如衰弱指数）识别加速衰老的中年人，可能有助于预防过早死亡及延长健康期望寿命。
