# Supplementary material for: Frailty index and all-cause and cause-specific mortality in Chinese adults: a prospective cohort study
Source: Lancet Public Health. 2020 Nov 30;5(12):e650–60. doi: 10.1016/S2468-2667(20)30113-4 (PMC7708389; doi:10.1016/S2468-2667(20)30113-4)
Supplement: Supplementary appendix 2 [file mmc2.pdf]

THE LANCET
Public Health

Supplementary appendix 2

This appendix formed part of the original submission and has been peer reviewed. We post it as supplied by the authors.

Supplement to: FanJ, YuC, Guo Y, et al. Frailty index and all-cause and cause-specific mortality in Chinese adults: a prospective cohort study. *Lancet Public Health* 2020; **5:** e650–60.

Appendix Members of the China Kadoorie Biobank collaborative group

International Steering Committee: Junshi Chen, Zhengming Chen (PI), Robert Clarke, Rory Collins, Yu Guo, Liming Li (PI), Jun Lv, Richard Peto, Robin Walters. **International Co-ordinating Centre, Oxford:** Daniel Avery, Ruth Boxall, Derrick Bennett, Yumei Chang, Yiping Chen, Zhengming Chen, Robert Clarke, Huaidong Du, Simon Gilbert, Alex Hacker, Mike Hill, Michael Holmes, Andri Iona, Christiana Kartsonaki, Rene Kerosi, Ling Kong, Om Kurmi, Garry Lancaster, Sarah Lewington, Kuang Lin, John McDonnell, Iona Millwood, Qunhua Nie, Jayakrishnan Radhakrishnan, Paul Ryder, Sam Sansome, Dan Schmidt, Paul Sherliker, Rajani Sohoni, Becky Stevens, Iain Turnbull, Robin Walters, Jenny Wang, Lin Wang, Neil Wright, Ling Yang, Xiaoming Yang. **National Co-ordinating Centre, Beijing:** Zheng Bian, Yu Guo, Xiao Han, Can Hou, Jun Lv, Pei Pei, Chao Liu, Canqing Yu. **10 Regional Co-ordinating Centres: Qingdao CDC:** Zengchang Pang, Ruqin Gao, Shanpeng Li, Shaojie Wang, Yongmei Liu, Ranran Du, Yajing Zang, Liang Cheng, Xiaocao Tian, Hua Zhang, Yaoming Zhai, Feng Ning, Xiaohui Sun, Feifei Li. **Licang CDC:** Silu Lv, Junzheng Wang, Wei Hou. **Heilongjiang Provincial CDC:** Mingyuan Zeng, Ge Jiang, Xue Zhou. **Nangang CDC:** Liqiu Yang, Hui He, Bo Yu, Yanjie Li, Qinai Xu,Quan Kang, Ziyan Guo. **Hainan Provincial CDC:** Dan Wang, Ximin Hu, Jinyan Chen, Yan Fu, Zhenwang Fu, Xiaohuan Wang. **Meilan CDC:** Min Weng, Zhendong Guo, Shukuan Wu,Yilei Li, Huimei Li, Zhifang Fu. **Jiangsu Provincial CDC:** Ming Wu, Yonglin Zhou, Jinyi Zhou, Ran Tao, Jie Yang, Jian Su. **Suzhou CDC:** Fang liu, Jun Zhang, Yihe Hu, Yan Lu, , Liangcai Ma, Aiyu Tang, Shuo Zhang, Jianrong Jin, Jingchao Liu. **Guangxi Provincial CDC:** Zhenzhu Tang, Naying Chen, Ying Huang. **Liuzhou CDC:** Mingqiang Li, Jinhuai Meng, Rong Pan, Qilian Jiang, Jian Lan,Yun Liu, Liuping Wei, Liyuan Zhou, Ningyu Chen Ping Wang, Fanwen Meng, Yulu Qin,, Sisi Wang. **Sichuan Provincial CDC:** Xianping Wu, Ningmei Zhang, Xiaofang Chen,Weiwei Zhou. **Pengzhou CDC:** Guojin Luo, Jianguo Li, Xiaofang Chen, Xunfu Zhong, Jiaqiu Liu, Qiang Sun. **Gansu Provincial CDC:** Pengfei Ge, Xiaolan Ren, Caixia Dong. **Maiji CDC:** Hui Zhang, Enke Mao, Xiaoping Wang, Tao Wang, Xi zhang. **Henan Provincial CDC:** Ding Zhang, Gang Zhou, Shixian Feng, Liang Chang, Lei Fan. **Huixian CDC:** Yulian Gao, Tianyou He, Huarong Sun, Pan He, Chen Hu, Xukui Zhang, Huifang Wu, Pan He. **Zhejiang Provincial CDC:** Min Yu, Ruying Hu, Hao Wang. Tongxiang CDC: Yijian Qian, Chunmei Wang, Kaixu Xie, Lingli Chen, Yidan Zhang, Dongxia Pan, Qijun Gu. **Hunan Provincial CDC:** Yuelong Huang, Biyun Chen, Li Yin, Huilin Liu, Zhongxi Fu, Qiaohua Xu. **Liuyang CDC:** Xin Xu, Hao Zhang, Huajun Long, Xianzhi Li, Libo Zhang, Zhe Qiu.

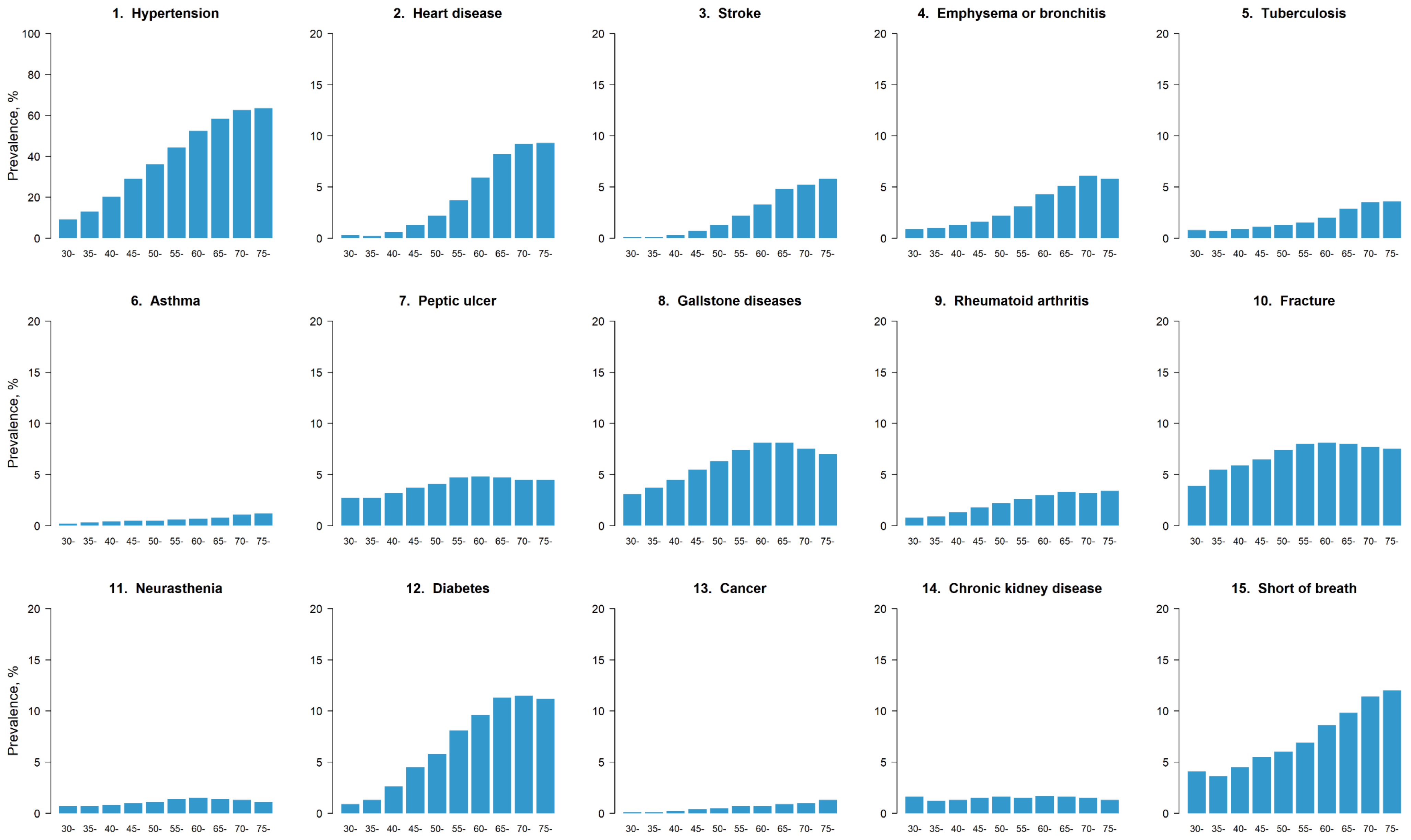
1. Hypertension
2. Heart disease
3. Stroke
4. Emphysema or bronchitis
5. Tuberculosis
6. Asthma
7. Peptic ulcer
8. Gallstone diseases
9. Rheumatoid arthritis
10. Fracture
11. Neurasthenia
12. Diabetes
13. Cancer
14. Chronic kidney disease
15. Short of breath
Prevalence, %
30- 35- 40- 45- 50- 55- 60- 65- 70- 75-

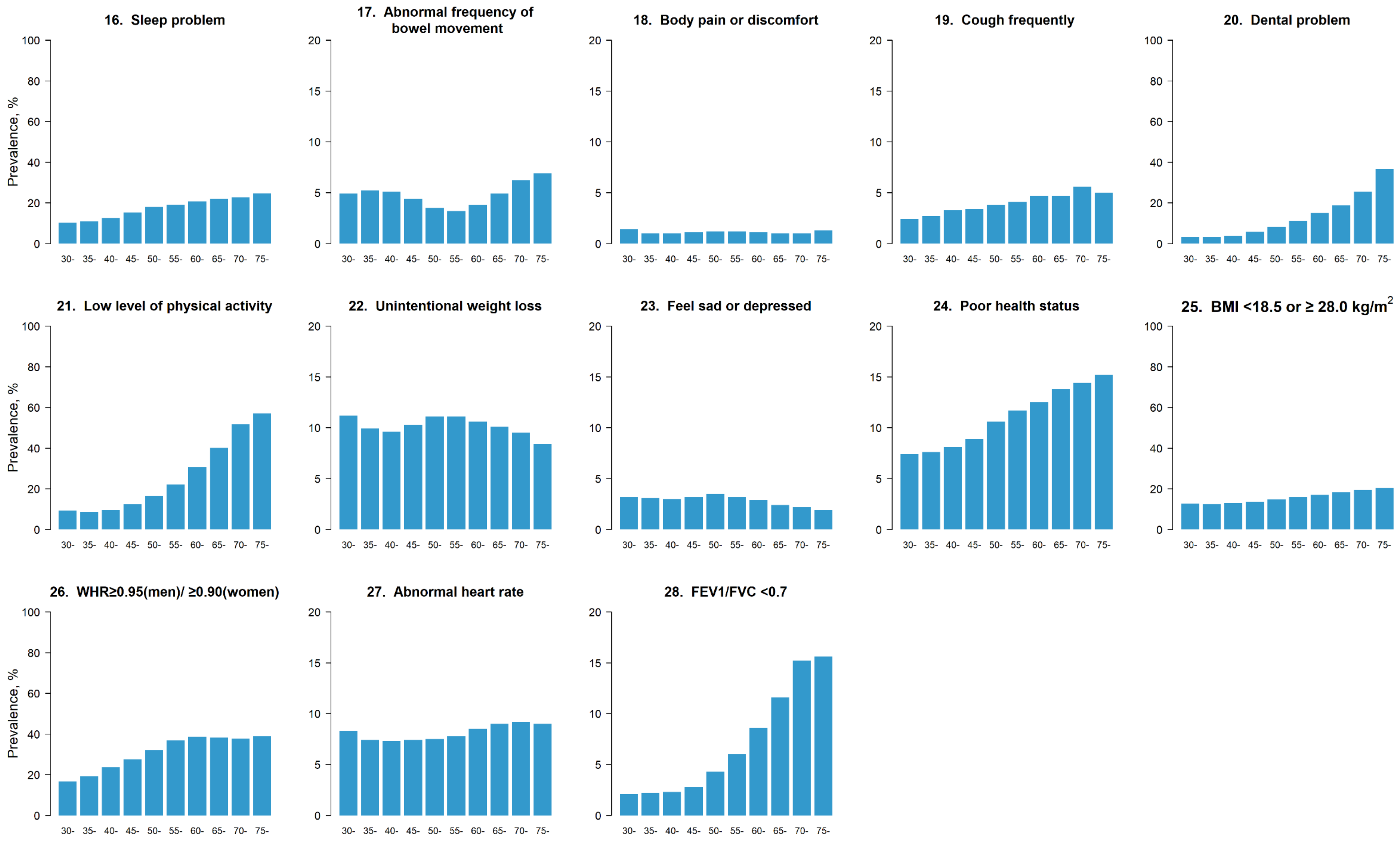
16. Sleep problem
17. Abnormal frequency of bowel movement
18. Body pain or discomfort
19. Cough frequently
20. Dental problem
21. Low level of physical activity
22. Unintentional weight loss
23. Feel sad or depressed
24. Poor health status
25. BMI <18.5 or ≥ 28.0 kg/m2
26. WHR≥0.95(men)/ ≥0.90(women)
27. Abnormal heart rate
28. FEV1/FVC <0.7
Prevalence, %
30- 35- 40- 45- 50- 55- 60- 65- 70- 75-

Figure S1 Prevalence of 28 deficits by age at baseline
BMI=body mass index; WHR=waist hip ratio; FEV1=forced expiratory volume in 1 s; FVC= forced vital capacity

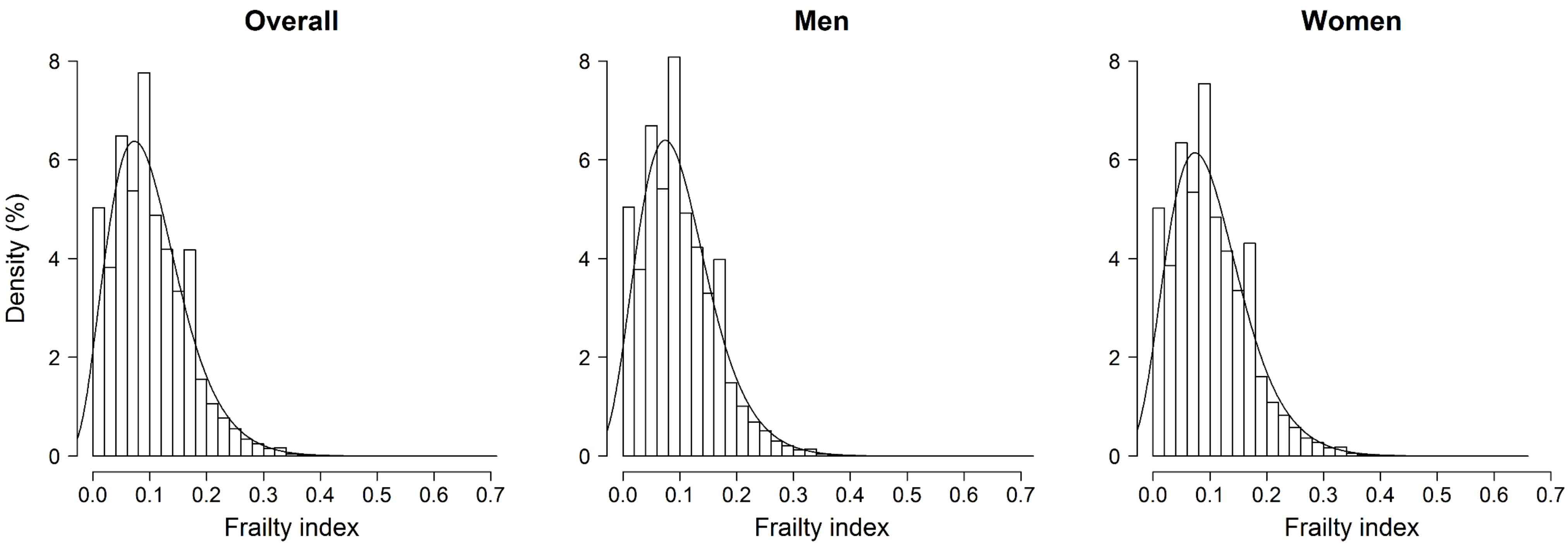

Figure S2 Distribution of FI at baseline

The solid line represents the fitted density plot of the FI.

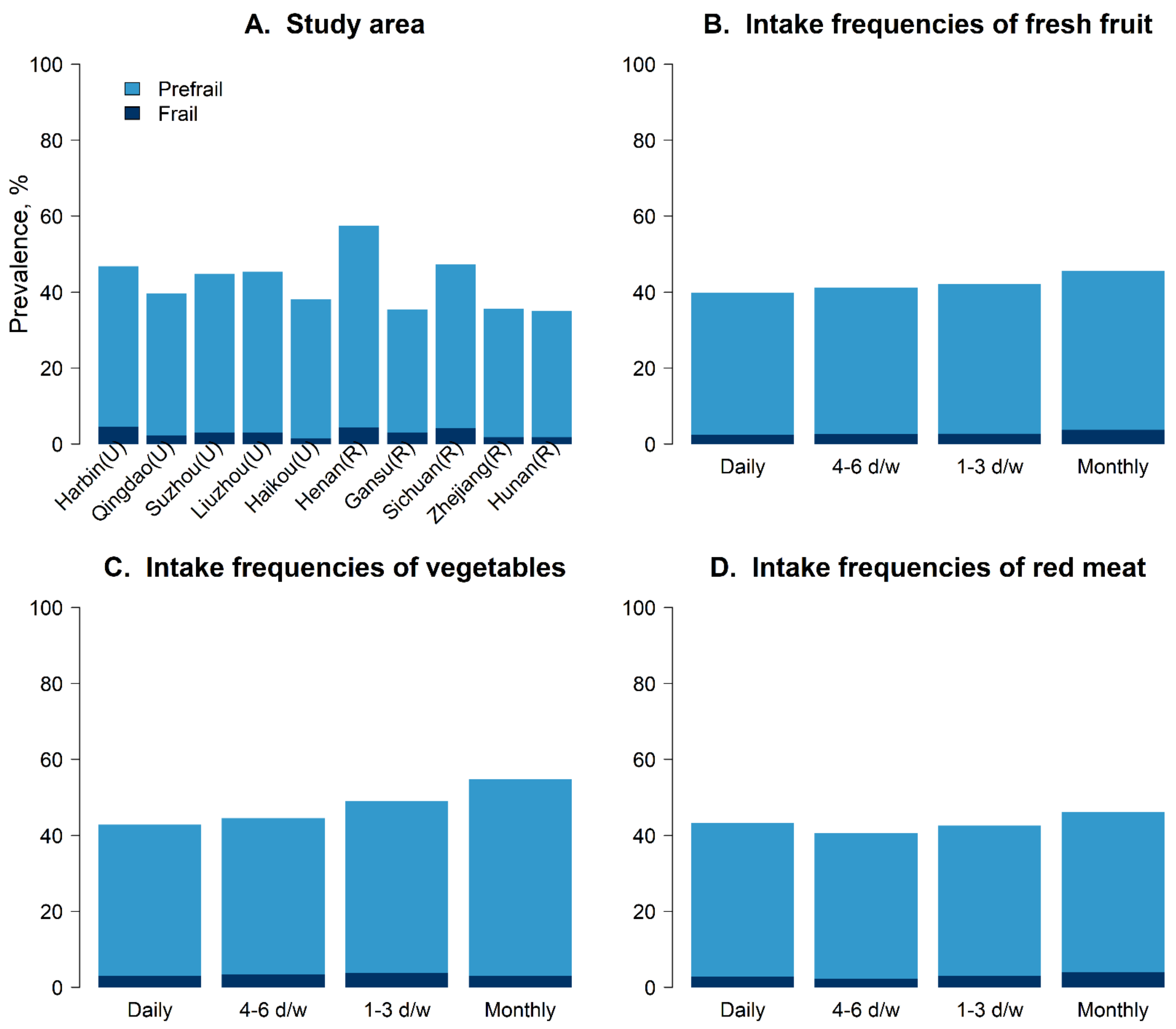
A. Study area
B. Intake frequencies of fresh fruit
C. Intake frequencies of vegetables
D. Intake frequencies of red meat
Prefrail
Frail
Prevalence, %
0
20
40
60
80
100
Harbin(U)
Qingdao(U)
Suzhou(U)
Liuzhou(U)
Haikou(U)
Henan(R)
Gansu(R)
Sichuan(R)
Zhejiang(R)
Hunan(R)
Daily
4-6 d/w
1-3 d/w
Monthly

Figure S3 Frailty prevalence by study area and dietary habits

Frailty prevalence was adjusted for age, sex, and study area, except that by study area. The P values for trend by intake frequencies of fresh fruit, vegetables, and red meat was <0.0001, 0.054, and <0.0001, respectively.

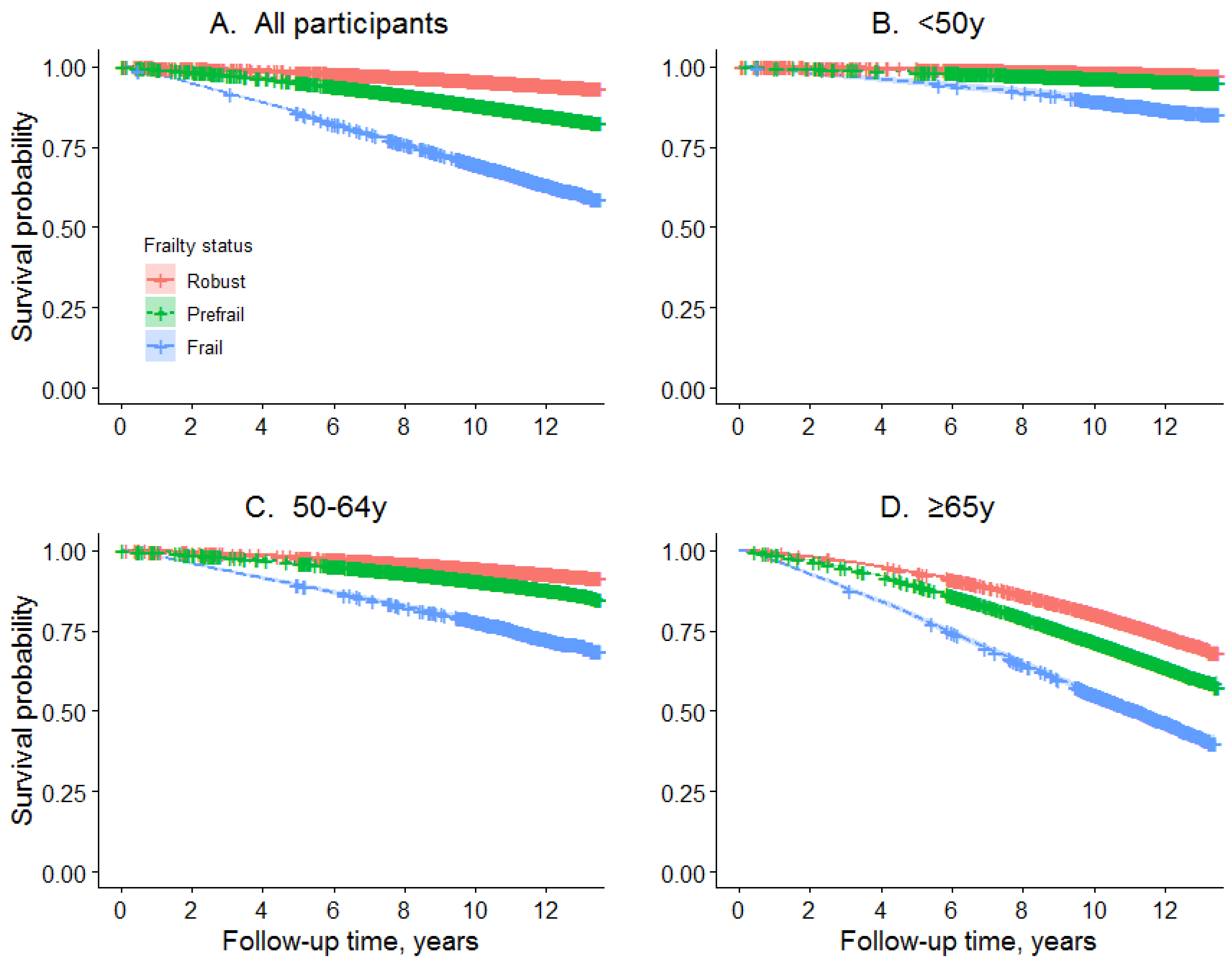
A. All participants
B. <50y
C. 50-64y
D. ≥65y
Survival probability
1.00
0.75
0.50
0.25
0.00
0
2
4
6
8
10
12
Follow-up time, years
Frailty status
Robust
Prefrail
Frail

Figure S4 Kaplan Meier survival probabilities by frailty status stratified by baseline age

All p-values for the log-rank test comparing survival distributions of three frailty status groups were < 0.0001.

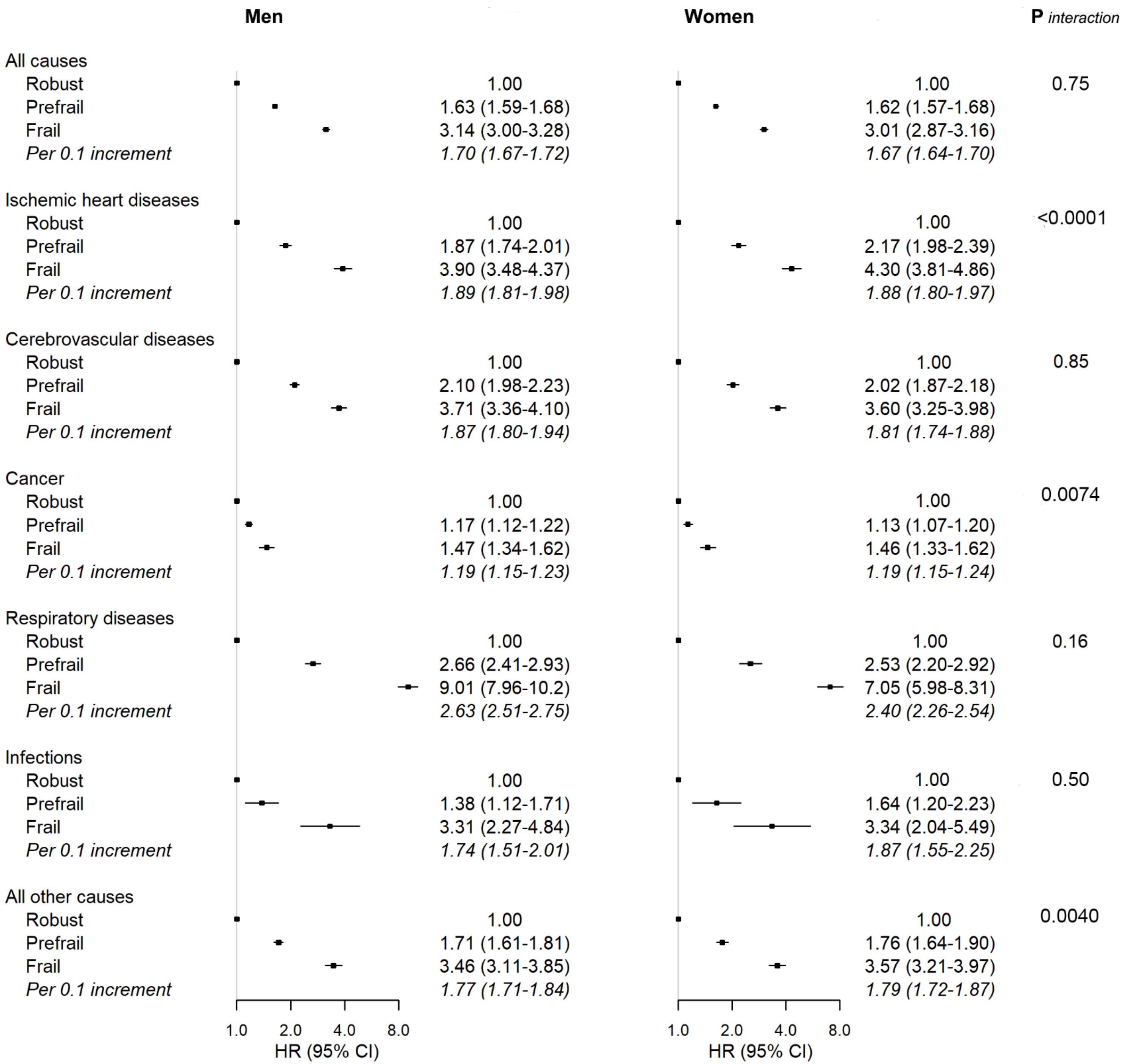

Men
Women
P interaction
All causes
Robust 1.00 1.00 0.75
Prefrail 1.63 (1.59-1.68) 1.62 (1.57-1.68)
Frail 3.14 (3.00-3.28) 3.01 (2.87-3.16)
Per 0.1 increment 1.70 (1.67-1.72) 1.67 (1.64-1.70)
Ischemic heart diseases
Robust 1.00 1.00 <0.0001
Prefrail 1.87 (1.74-2.01) 2.17 (1.98-2.39)
Frail 3.90 (3.48-4.37) 4.30 (3.81-4.86)
Per 0.1 increment 1.89 (1.81-1.98) 1.88 (1.80-1.97)
Cerebrovascular diseases
Robust 1.00 1.00 0.85
Prefrail 2.10 (1.98-2.23) 2.02 (1.87-2.18)
Frail 3.71 (3.36-4.10) 3.60 (3.25-3.98)
Per 0.1 increment 1.87 (1.80-1.94) 1.81 (1.74-1.88)
Cancer
Robust 1.00 1.00 0.0074
Prefrail 1.17 (1.12-1.22) 1.13 (1.07-1.20)
Frail 1.47 (1.34-1.62) 1.46 (1.33-1.62)
Per 0.1 increment 1.19 (1.15-1.23) 1.19 (1.15-1.24)
Respiratory diseases
Robust 1.00 1.00 0.16
Prefrail 2.66 (2.41-2.93) 2.53 (2.20-2.92)
Frail 9.01 (7.96-10.2) 7.05 (5.98-8.31)
Per 0.1 increment 2.63 (2.51-2.75) 2.40 (2.26-2.54)
Infections
Robust 1.00 1.00 0.50
Prefrail 1.38 (1.12-1.71) 1.64 (1.20-2.23)
Frail 3.31 (2.27-4.84) 3.34 (2.04-5.49)
Per 0.1 increment 1.74 (1.51-2.01) 1.87 (1.55-2.25)
All other causes
Robust 1.00 1.00 0.0040
Prefrail 1.71 (1.61-1.81) 1.76 (1.64-1.90)
Frail 3.46 (3.11-3.85) 3.57 (3.21-3.97)
Per 0.1 increment 1.77 (1.71-1.84) 1.79 (1.72-1.87)
1.0 2.0 4.0 8.0
HR (95% CI)
1.0 2.0 4.0 8.0
HR (95% CI)

Figure S5 Association of frailty status with total and cause-specific mortality in men and women

All models were stratified by age and study area. Multivariate models were adjusted for the same covariates as in the Figure 3.

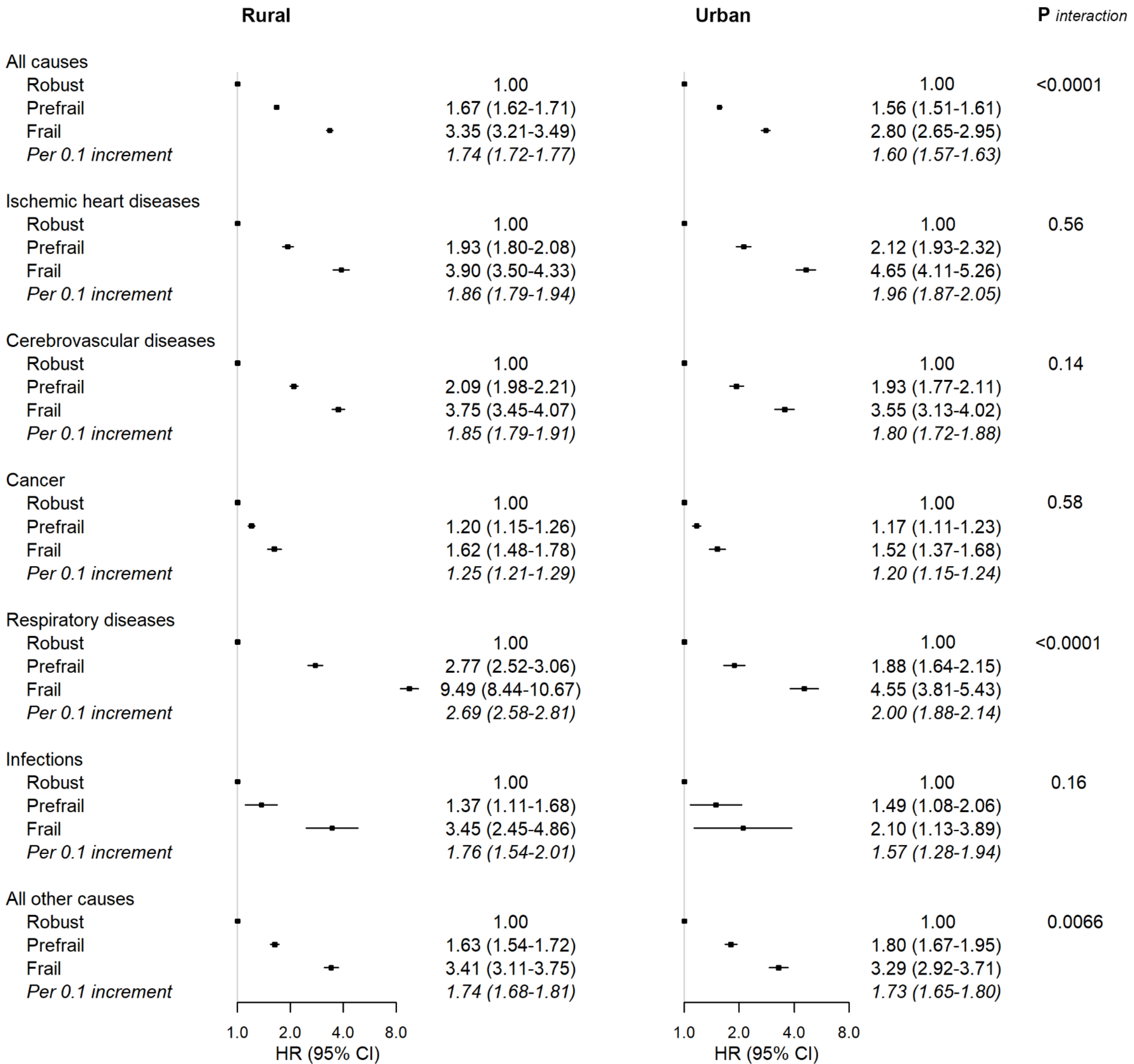
Rural
Urban
P interaction
All causes
Robust 1.00 1.00 <0.0001
Prefrail 1.67 (1.62-1.71) 1.56 (1.51-1.61)
Frail 3.35 (3.21-3.49) 2.80 (2.65-2.95)
Per 0.1 increment 1.74 (1.72-1.77) 1.60 (1.57-1.63)
Ischemic heart diseases
Robust 1.00 1.00 0.56
Prefrail 1.93 (1.80-2.08) 2.12 (1.93-2.32)
Frail 3.90 (3.50-4.33) 4.65 (4.11-5.26)
Per 0.1 increment 1.86 (1.79-1.94) 1.96 (1.87-2.05)
Cerebrovascular diseases
Robust 1.00 1.00 0.14
Prefrail 2.09 (1.98-2.21) 1.93 (1.77-2.11)
Frail 3.75 (3.45-4.07) 3.55 (3.13-4.02)
Per 0.1 increment 1.85 (1.79-1.91) 1.80 (1.72-1.88)
Cancer
Robust 1.00 1.00 0.58
Prefrail 1.20 (1.15-1.26) 1.17 (1.11-1.23)
Frail 1.62 (1.48-1.78) 1.52 (1.37-1.68)
Per 0.1 increment 1.25 (1.21-1.29) 1.20 (1.15-1.24)
Respiratory diseases
Robust 1.00 1.00 <0.0001
Prefrail 2.77 (2.52-3.06) 1.88 (1.64-2.15)
Frail 9.49 (8.44-10.67) 4.55 (3.81-5.43)
Per 0.1 increment 2.69 (2.58-2.81) 2.00 (1.88-2.14)
Infections
Robust 1.00 1.00 0.16
Prefrail 1.37 (1.11-1.68) 1.49 (1.08-2.06)
Frail 3.45 (2.45-4.86) 2.10 (1.13-3.89)
Per 0.1 increment 1.76 (1.54-2.01) 1.57 (1.28-1.94)
All other causes
Robust 1.00 1.00 0.0066
Prefrail 1.63 (1.54-1.72) 1.80 (1.67-1.95)
Frail 3.41 (3.11-3.75) 3.29 (2.92-3.71)
Per 0.1 increment 1.74 (1.68-1.81) 1.73 (1.65-1.80)
1.0 2.0 4.0 8.0
HR (95% CI)
1.0 2.0 4.0 8.0
HR (95% CI)

Figure S6 Association of frailty status with total and cause-specific mortality in rural and urban area

All models were stratified by age and sex. Multivariate models were adjusted for the same covariates as in the Figure 3.

Table S1 Sensitivity analyses for association of per 0.1 increment of FI with all-cause and cause-specific mortality

	Primary results	Removing participants with corresponding diseases at baseline						Removing current smokers[†]	Removing death during the first 5 years of follow-up
		Heart diseases	Stroke or TIA	Cancer	COPD[*]	Diabetes	Any of the above		
Number of participants	512,723	497,251	503,839	510,145	475,666	482,423	428,435	361,920	496,074
Mean FI (SD)	0.099 (0.064)	0.100 (0.064)	0.101 (0.065)	0.103 (0.066)	0.102 (0.065)	0.098 (0.063)	0.109 (0.068)	0.099 (0.064)	0.098 (0.063)
All-cause mortality	1.68 (1.66-1.71)	1.69 (1.67-1.71)	1.62 (1.60-1.64)	1.64 (1.62-1.67)	1.61 (1.59-1.63)	1.60 (1.58-1.62)	1.36 (1.34-1.38)	1.69 (1.66-1.72)	1.61 (1.58-1.63)
Cause-specific mortality									
Ischemic heart diseases	1.89 (1.83-1.94)	1.81 (1.75-1.88)	1.82 (1.77-1.88)	1.84 (1.79-1.90)	1.91 (1.85-1.98)	1.76 (1.70-1.83)	1.56 (1.49-1.63)	1.88 (1.81-1.96)	1.79 (1.72-1.85)
Cerebrovascular diseases	1.84 (1.79-1.89)	1.88 (1.83-1.93)	1.65 (1.60-1.69)	1.81 (1.76-1.85)	1.92 (1.87-1.97)	1.76 (1.72-1.81)	1.58 (1.53-1.63)	1.84 (1.78-1.90)	1.76 (1.70-1.81)
Cancer	1.19 (1.16-1.22)	1.20 (1.17-1.23)	1.20 (1.17-1.23)	1.14 (1.11-1.17)	1.16 (1.13-1.19)	1.18 (1.15-1.21)	1.09 (1.06-1.12)	1.20 (1.16-1.24)	1.13 (1.09-1.16)
Respiratory diseases	2.54 (2.45-2.63)	2.66 (2.56-2.76)	2.57 (2.48-2.66)	2.46 (2.38-2.55)	1.77 (1.67-1.86)	2.62 (2.53-2.72)	1.67 (1.57-1.78)	2.48 (2.35-2.61)	2.31 (2.21-2.42)
Infections	1.78 (1.59-2.00)	1.80 (1.60-2.02)	1.78 (1.59-2.00)	1.76 (1.58-1.96)	1.57 (1.38-1.79)	1.73 (1.53-1.95)	1.44 (1.26-1.65)	1.82 (1.55-2.13)	1.65 (1.41-1.93)
All other causes	1.78 (1.73-1.83)	1.79 (1.74-1.84)	1.74 (1.69-1.79)	1.75 (1.71-1.80)	1.78 (1.73-1.84)	1.52 (1.47-1.57)	1.41 (1.36-1.46)	1.81 (1.74-1.88)	1.72 (1.66-1.78)

FI: frailty index; COPD: chronic obstructive pulmonary disease.

[*]COPD was defined as self-reported doctor diagnosis of emphysema or chronic bronchitis, or the ratio of measured forced expiratory volume in 1 s over measured forced vital capacity (FEV1/FVC) <0.7.

[†]Daily smokers and former smokers who quitted smoking due to illness.
